# Supplementary material for: The use of quarantine as an international travel measure during the COVID-19 pandemic: A comparative analysis of implementation and equity impacts in five “exemplar” countries
Source: PLOS Glob Public Health. 2025 Nov 14;5(11):e0005457. doi: 10.1371/journal.pgph.0005457 (PMC12617841; doi:10.1371/journal.pgph.0005457)
Supplement: S1 Annex — (DOCX) [file pgph.0005457.s001.docx]

**S1 Annex: Legal framework for quarantine use during the COVID-19 pandemic in the five countries**

| **Country** | What type of political system does each country have?  What laws provide the authority to apply quarantine requirements?  What is the stated purpose of the legal framework?  What is the strength and scope of authority provided by the legislation? |
| --- | --- |
| Australia | Australia is a representative democracy and a constitutional monarchy. It is also a federation of states.  **Biosecurity Act (2015)**  The Commonwealth (Federal) Government holds authority to adopt quarantine requirements related to international borders which are set out in the Biosecurity Act (2015). The Commonwealth Government announced “enhanced border control measures'' on February 1 2020 as part of its plan to evacuate Australian citizens and residents from China and quarantine them in a detention centre on Christmas Island for 14 days prior to arrival in Australia. This was done under discretionary powers granted by the National Security Committee, made possible as a result of the addition of “human coronavirus with pandemic potential” to the Biosecurity (Listed Human Diseases) Determination 2016.^^[[1]](#endnote-1)^^ It was followed by subsequents evacuations with on-shore quarantine. It exercised human biosecurity emergency (HBE) powers under the Act for the first time on 15 March 2020, mandating 14 day quarantine and closing international borders from 19 March 2020.^^[[2]](#endnote-2)^^  **Amendments to State/Territory Public Health Acts**  Implementation of quarantine for travellers as a public health measure, as stipulated by the Commonwealth Government, was the responsibility of states and territories. There is a longstanding lack of clarity about how HBE and state/territorial public health powers intersect. During COVID-19, each state/territory was required to apply their own legislative arrangements regarding biosecurity, resulting in individual rules and arrangements for quarantine. Amendments to state/territorial Public Health Acts were adopted in some cases. |
| Aotearoa New Zealand | Aotearoa New Zealand is a representative democracy and a constitutional monarchy.  **Health Act (1956, amended 2006)**  Section 97   1. A person is liable to quarantine if he or she is on board, or disembarks from, a craft that is liable to quarantine. 2. This subsection applies to a person liable to quarantine if the medical officer of health believes or suspects, on reasonable grounds,— 3. that he or she is infected with a quarantinable disease; or 4. that, within the 14 days before he or she arrived in New Zealand, he or she has been exposed to a disease that (whether or not it was a quarantinable disease at the time of the believed or suspected exposure) is a quarantinable disease.   Section 118  Regulations made under this Act may provide for all or any of the following matters:   1. the procedure to be adopted in the inspection of ships or aircraft arriving in New Zealand and in the examination of persons on such ships or arriving by such aircraft; the conditions subject to which pratique may be granted; and generally the performance of quarantine: 2. the measures of disinfection, including the disinfection of any things, to be adopted in respect of any ship or aircraft on which any infectious disease exists or is reported to exist: 3. the isolation and treatment of persons arriving in New Zealand who are or are suspected to be suffering from any quarantinable disease or who have or are suspected to have been exposed to the infection of a quarantinable disease or who are otherwise liable to quarantine under Section 97:^^[[3]](#endnote-3)^^    1. the payment by any person who has been isolated pursuant to regulations made under this section of the reasonable cost of his treatment and maintenance while in isolation, and the exemption of any person, in whole or in part, from liability to make such payment:  the release of persons from quarantine, either unconditionally or subject to conditions as to medical surveillance or otherwise:the exemption of ships, aircraft, persons, or things of any specified classes, or in any specified circumstances or classes of circumstances, from the operation of any of the provisions of Part 4^^[[4]](#endnote-4)^^ or of any regulations under this Act, either wholly or in part or subject to conditions:the powers, functions, and duties of medical officers of health and other officers in respect of quarantine or of any of the aforesaid matters. **COVID-19 Public Health Response Act (2020)**  This Act provides the legal framework for responding to COVID-19 and allows the Minister for COVID-19 Response to make orders under section 11 to give effect to the public health response. Sections 32P-R concern managed isolation and quarantine facility or other places of isolation or quarantine.  **COVID19 Amendment Act (2022)**  This Act provides the legal framework for public health measures following the emergency phase of the pandemic response. The Act recognizes that Managed Isolation and Quarantine is no longer needed. |
| Singapore | Singapore is a representative democratic republic with political authority entrusted to a unitary parliament and central government which holds all ultimate authority.  **Infectious Diseases Act (1976)**   - The main legislation for the prevention and control of infectious diseases, the Act is jointly administered by the Ministry of Health and the National Environment Agency (NEA), under the purview of the Director-General of Health (Ministry of Health) and the Director-General of Food Administration (now under Singapore Food Agency). - “For the prevention of the introduction of infectious diseases into Singapore, the IDA allows the Minister to declare an area (whether in Singapore or elsewhere) to be an infected area if there is reason to believe that a dangerous infectious disease may be introduced into Singapore through or from that area. The Director-General Public Health is empowered to stipulate the necessary measures to be taken to prevent the introduction or importation of infectious diseases into Singapore through its ports of entry.” - 15: “(1)  The Director-General of Health may order any person who is, or is suspected to be, a case or carrier or contact of an infectious disease to be detained and isolated in a hospital or other place for such period of time and subject to such conditions as the Director-General of Health may determine.” - The IDA does not empower the Director-Generals to impose control orders to restrict movements of individuals, enforce control orders or appoint public officers to undertake enforcement of containment measures.   **COVID-19 (Temporary Measures) Act 2020**  Part 7 (COVID-19 Control Orders)   - The Act empowers the Health Minister to adopt regulations or impose control orders for “the purpose of preventing, protecting against, delaying or otherwise controlling the incidence or transmission of COVID-19 in Singapore” - Requires quarantine of individuals [Article 34:2A: to require people or certain people to stay at or in, and not leave, a specified place (whether or not a place of accommodation)] - The contravention of a quarantine or control order is considered an offence that is liable to conviction (fine of S$10,000 or imprisonment not exceeding 6 months or both for first-time offences). |
| South Korea^^[[5]](#endnote-5)^^ | The Republic of Korea is a representative democratic republic with an elected unicameral legislature.  **Infectious Disease Control and Prevention Act (1957, Amended 2023)**  The Act, administered by the Ministry of Health and Welfare and Korean Disease Control and Prevention Agency (KDCA), provides the legal basis for quarantine. It specifies detailed procedures such as quarantine periods, location, cost, and penalty through administrative order under the Act.  **Quarantine Act (1954, Amended 2017)**   - The Act, administered by the KDCA, provides for monitoring and screening procedures at POEs, including countries that require monitoring. - The purpose is to prevent the spread of infectious diseases within the Republic of Korea and overseas by providing for matters concerning the procedures for quarantining persons, all means of transportation and cargo, which enter or depart, and measures for preventing infectious diseases, thereby contributing to the maintenance and protection of public health.   Article 5.2  The Minister of Health and Welfare may take quarantine measures against a person who has ever stayed in, or travelled via, an adjacent area to a contaminated area designated under Article 5 (1) where a quarantinable infectious disease could break out (hereinafter referred to as “adjacent area to a contaminated area”) by requiring to submit a health condition questionnaire, checking whether the person has a fever, or taking other measures if necessary to prevent the quarantinable infectious disease. |
| Taiwan | Taiwan is a representative democratic republic with an elected unicameral legislature and President.  **Communicable Disease Control Act  (1944, Amended 2023)**  Article 58  Competent authorities may impose the following quarantine or measures on persons entering, exiting the country (border), and may collect associated fees:   1. provide quarantine information, anti-disease drugs, immunization, or issue warnings to persons visiting epidemic areas; 2. in accordance with regulations set by the central competent authority, carefully and accurately fill out and submit the communicable disease report forms and tables, and depending on the actual situation, present health certificates or other relevant certificates; 3. conduct health assessment or impose other quarantine measures; 4. impose home-based quarantine, group quarantine, isolation care or other necessary measures on persons entering from affected areas, contacts or suspected contacts, patients or suspected patients with communicable disease; 5. inform immigration authorities to restrict patients who have not been fully cured and are expected to infect others when exiting the country (border); 6. request organizations concerned to stop issuing permits for entering the country (border) to persons of certain countries or areas or providing other assistance.   For persons mentioned in the preceding Subparagraph 5, when no longer capable of infecting others, competent authorities shall immediately inform the immigration authorities to abolish their exit restrictions.  To the quarantine or the measures mentioned in Paragraph 1 enforced by competent authorities, persons entering or exiting the country (border) shall not refuse, evade or obstruct. |

1. Australia. *Biosecurity Act 2015*. 9 April 2020. <https://www.legislation.gov.au/C2015A00061/2020-03-25/text> [↑](#endnote-ref-1)
2. Morrison S. Coronavirus measures endorsed by National Cabinet. Media Release, Parliament of Australia, 16 March 2020. <https://parlinfo.aph.gov.au/parlInfo/search/display/display.w3p;query=Id%3A%22media%2Fpressrel%2F7245039%22> [↑](#endnote-ref-2)
3. New Zealand Parliamentary Counsel Office. *Health Act 1956*, No. 65, Section 97, People liable to quarantine, 19 December 2006. <https://www.legislation.govt.nz/act/public/1956/0065/latest/DLM307464.html#DLM307464> [↑](#endnote-ref-3)
4. New Zealand Parliamentary Counsel Office. *Health Act 1956*, No. 65, Part 4 Quarantine, 1 April 1983. <https://www.legislation.govt.nz/act/public/1956/0065/latest/DLM307455.html#DLM307455> [↑](#endnote-ref-4)
5. Kwon S, Lee H, Ki M, Chung DW, Baris E. *Republic of Korea’s COVID-19 Preparedness and Response*. World Bank Group Korea Office, Innovation and Technology Note Series, November 2020. <https://documents1.worldbank.org/curated/en/534981606291035033/pdf/Republic-of-Korea-s-COVID-19-Preparedness-and-Response.pdf> [↑](#endnote-ref-5)
