# Supplementary material for: The use of quarantine as an international travel measure during the COVID-19 pandemic: A comparative analysis of implementation and equity impacts in five “exemplar” countries
Source: PLOS Glob Public Health. 2025 Nov 14;5(11):e0005457. doi: 10.1371/journal.pgph.0005457 (PMC12617841; doi:10.1371/journal.pgph.0005457)
Supplement: S2 Annex — (DOCX) [file pgph.0005457.s002.docx]

**S2 Annex: Timing of quarantine use by the five countries**

| Australia | - 1 February 2020: Nationals and residents arriving on evacuation flights from Hubei Province, China are required to quarantine on Christmas Island (offshore territory) for 14 days. - 1 February 2020:  International arrivals travelling from China, who are non-nationals or non-residents, prohibited from entering country. Nationals and permanent residents travelling from China are required to quarantine at home for 14 days. - 15 March 2020:  International arrivals must quarantine at home for 14 days. - 20 March 2020: Non-nationals and non-residents prohibited from entering Australia. - 27 March 2020:  Returning nationals and residents must quarantine in designated facility (usually hotel) for 14 days. - 27 March 2020-1 November 2021: Quarantine requirements continuous for returning nationals and residents, with very few exceptions. - 16 October 2020: Arrivals from New Zealand (regardless of citizenship or residence) permitted to enter without mandatory quarantine if they have been in New Zealand for at least 14 days. Arrangement partially suspended from January 25-31, February 15–21, and February 25–March 20, 2021 due to community transmission in New Zealand. - 1 November 2021:  NSW and Victoria lift quarantine requirements for vaccinated inbound nationals or residents - 27 November 2021:  In response to South Africa’s isolation of the Omicron variant, nationals or residents arriving in any state and territory from 9 African countries are required to quarantine in designated hotels for 14 days.  Travellers recently arrived from those countries must quarantine at home for 14 days from the date of arrival. - 27 November 2021: ACT requires inbound nationals or residents not arriving from “countries of concern” to quarantine at home until November 30. NSW and Victoria require inbound nationals or residents not arriving from “countries of concern” to quarantine at home for 72 hours. - 1 December 2021:  NSW reintroduces quarantine at home (3 days) for international arrivals, with PCR testing upon arrival and on day 6. - 15 December 2021: Quarantine requirements aimed at 9 African countries and Omicron variant lifted for vaccinated inbound nationals or residents. - 15 December 2021:  International arrivals who are non-nationals and non-residents, who are student visa holders, skilled workers and other designated categories of travellers, permitted to enter with proof of vaccination and negative test. Need to quarantine dependent on the state/territory. - 21 February 2022:  International arrivals who are non-nationals and non-residents permitted to enter with proof of vaccination. All states and territories “quarantine-free” except Western Australia.^^[[1]](#endnote-1)^^ - 21 February 2022:  NSW and Victoria shorten quarantine requirement in designated hotel from 14 to 7 days for unvaccinated international arrivals (nationals and residents). - 1 June 2022: Unvaccinated international arrivals who are nationals and residents are permitted to enter all states and territories without quarantine requirement. |
| --- | --- |
| Aotearoa New Zealand^^[[2]](#endnote-2)^^ | - 2 Feb 2020: New Zealanders repatriated from Wuhan quarantined for 14 days at isolation centre - 16 March 2020:  14 days ‘self-isolation’ / home quarantine “expected” for international arrivals - 19 March 2020: International border closed to non-citizens and residents and all arrivals were “required” to quarantine at home. - 9 April 2020: Mandatory supervised 14-day quarantine “Managed Isolation” (MIQ) for international arrivals^^[[3]](#endnote-3)^^ - 11 Aug: Charges introduced to partially cover MIQ costs - 5 October 2020: Managed Isolation Allocation System introduced to manage demand for scarce MIQ facilities. From 3 November incoming travelers are legally required to secure a place in MIQ as a condition of air travel. - 21 January 2021: Travellers from Cook Islands exempt from MIQ - 24 March 2021: Travellers from Niue exempt from MIQ - 19 April - July (different dates for different states) 2021: Quarantine-free travel bubble between New Zealand and Australia - September 2021: Virtual online lobby system introduced to manage MIQ demand (functions like a lottery, resets weekly) - Oct-Dec 2021 ‘Reconnecting New Zealand to the World’ scheme piloted, which saw some people allowed to isolate at home - 8 November 2021: quarantine free travel for all travelers from low-risk Pacific Islands - 14 November 2021: MIQ shortened to 7 days for international arrivals due to scarce hotel capacity, followed by isolation at home for three days. In December, following the introduction of the Omicron variant, the period was extended to “at least 10 days”. - Feb - March 2022:  International arrivals who are vaccinated are no longer required to quarantine in hotels, but requests to isolate at home for 10 (later changed to 7) days. Lifting of entry restrictions was staggered to allow different groups and visa classes to enter at different times - 3 May 2022: Citizens, PR, and visa holders who are unvaccinated are no longer required to quarantine - 31 July 2022: Vaccinated tourists and visa holders allowed quarantine-free entry - 12 September 2022: Unvaccinated tourists and visa holders allowed quarantine-free entry |
| Singapore | - 15 February 2020: All travellers returning to Singapore advised to monitor health closely for 2 weeks (14 days) upon return and seek medical attention promptly if unwell - 26 February 2020: Returning nationals, Permanent Residents (PRs) and long-term pass holders (including work passes and permits, Student’s Pass, Dependent’s Pass and Long-Term Visit Pass), with travel to Daegu and Cheongdo, South Korea in past 14 days, are subject to mandatory 14-day quarantine order or Stay-Home Notice (SHN). Non-nationals and non-PRs with travel to these regions are prohibited from entering the country. - 4 March 2020: Returning nationals, Permanent Residents (PRs) and long-term pass holders (including work passes and permits, Student’s Pass, Dependent’s Pass and Long-Term Visit Pass), with travel to Iran, northern Italy or Korea in past 14 days, are subject to mandatory 14-day quarantine order. Non-nationals and non-PRs with travel to these regions are prohibited from entering the country. - 15 March 2020: Returning nationals, Permanent Residents (PRs) and long-term pass holders (including work passes and permits, Student’s Pass, Dependent’s Pass and Long-Term Visit Pass), with travel to Italy, France, Spain and Germany in past 14 days, are subject to mandatory 14-day quarantine order. Non-nationals and non-PRs with travel to these regions are prohibited from entering the country. - 16 March 2020: Returning nationals, Permanent Residents (PRs) and long-term pass holders (including work passes and permits, Student’s Pass, Dependent’s Pass and Long-Term Visit Pass), with travel to ASEAN countries, Japan, Switzerland, UK in past 14 days, are subject to mandatory 14-day quarantine order. Proof of the place where they will quarantine is required. Non-nationals and non-PRs with travel to these regions are prohibited from entering the country. - 21 March 2020: A mandatory 14-day quarantine order issued for all international arrivals including Nationals and PRs, Long Term Pass holders and short-term visitors entering Singapore. Proof of the place where they will quarantine is required. - 23 March 2020-19 October 2021: Non-nationals and non-PRs prohibited from entering the country. - 25 March 2020: Mandatory 14-day quarantine in a designated facility (i.e. hotel) is required for all nationals, PRs, and long-term pass holders arriving from the US or UK. Transportation provided to transport travellers from the airport to quarantine hotels. - 6 April 2020: Mandatory 14-day quarantine in a designated facility (i.e. hotel) is required for all nationals, PRs, and long-term pass holders arriving from ASEAN countries, France, India and Switzerland. Transportation provided to transport travellers from the airport to quarantine hotels.   “Circuit Breaker” Period   - 7 April 2020-1 June 2020: Singapore introduced a nationwide lockdown for all residents of Singapore, including non-residents and closed its borders for short-term travellers. Only Singapore Citizens and Permanent Residents were allowed to re-enter Singapore and were subjected to a 14-day quarantine.   Phase 1 Reopening [2 June to 18 June 2020]   - 3 June 2020: “Fast Lane” arrangements allowed for international travel between Singapore and six Chinese provinces and municipalities (Shanghai, Tianjin, Chongjin, Guangdong, Jiangsu and Zhejiang) to facilitate essential business or official trips. Approved travellers must undergo a PCR test prior to and upon arrival, and then undergo 1-2 days of quarantine. If the upon arrival test is negative, the travelers may conduct their business in limited locations.^^[[4]](#endnote-4)^^ - 17 June 2020: Returning nationals, Permanent Residents (PRs) and long-term pass holders, who had remained in selected low risk countries/regions - Australia, Brunei Darussalam, Hong Kong, Japan, Macao, Mainland China, New Zealand, Republic of Korea, Taiwan and Vietnam) - for 14 days prior to entry, are allowed to quarantine at home rather than in a dedicated facility for 14 days. All other international arrivals must serve their 14-day quarantine in a dedicated facility.   Phase 2 Reopening [19 June to 27 December 2020]   - 20 July 2020: Returning nationals, Permanent Residents (PRs) and long-term pass holders, who had remained in Australia (except state of Victoria), Brunei Darussalam, Macao, Mainland China, New Zealand, Republic of Korea, Taiwan and Vietnam for 14 days prior to entry, are allowed to quarantine at home rather than in a dedicated facility for 14 days. All other international arrivals, including from Victoria (Australia), Japan and Hong Kong, must serve their 14-day quarantine in a dedicated facility. - 21 August 2020: Returning nationals, Permanent Residents (PRs) and long-term pass holders from Brunei Darussalam and New Zealand are subject to an upon arrival PCR test in lieu of quarantine. Arrivals from low-risk countries/regions - Australia (except Victoria state), Macao, Mainland China, Taiwan, Vietnam, Malaysia – are subject to a 7-day quarantine period at home. - 1 September 2020: Under the Air Travel Pass, non-nationals and non-residents from Brunei Darussalam and New Zealand are permitted to enter the country subject to an upon arrival PCR test in lieu of quarantine. Introduction of Air Travel Pass for Brunei and New Zealand to allow entry into Singapore.   Phase 3 Reopening [28 December 2020 to 7 May 2021]   - 19 January 2021: Returning nationals, Permanent Residents (PRs) and long-term pass holders from UK and South Africa, as high risk countries for the Omicron variant, required to undergo additional 7-day isolation at home following a 14-day quarantine at a dedicated facility. - 23 April 2021: Returning nationals, Permanent Residents (PRs) and long-term pass holders from India, as a high risk country for the Omicron variant, are required to undergo additional 7-day isolation at home following a 14-day quarantine at a dedicated facility. Migrant workers from India working in the Construction, Marine Shipyard, Process sectors and staying in dormitories are subject to a 21-day quarantine at a dedicated facility.   Reversion to Phase 2 Heightened Alert [8 May to 13 June 2021]   - 8 May 2021: Travel measures for international arrivals determined by travel history in the past 21 days instead of 14 days.   Phase 3 Heightened Alert [14 June to 21 July 2021] and Phase 2 Heightened Alert [22 July to 9 August 2021]   - 24 June 2021: All new travellers arriving from high-risk countries/regions subjected to a 14-day SHN instead of a 21-day SHN at dedicated SHN facilities. Travellers were also required to test themselves regularly with an ART self-test kit on Day 3, 7 and 11 of their arrival in Singapore while serving their SHN. This is on top of the COVID-19 PCR they have to take on arrival to Singapore and on Day 14 of their arrival before they exit SHN (ART implemented on 28 June 2021). Newly arrived migrant workers from higher risk countries and staying in dormitories or working in CMP sectors were required to undergo an additional 7-day stay at the Migrant Worker Onboarding Centre or a dedicated facility after they are cleared from their 14-day SHN at dedicated SHN facilities. The 7-day additional stay has been in place since early 2021 and will include an additional testing regime, medical examination and SIP.   Preparatory Stage of Transition [10 August to 26 August 2021]   - 19 August 2021: Countries classified by risk categories, each with different travel measures, based on a traveller’s 21-day travel history prior to entry.  1. Category 1 – Travellers permitted to enter subject to upon arrival PCR test. Travelers must quarantine while awaiting test results. If negative, the traveller may leave quarantine. 2. Category 2 – Travellers required to undergo 7-day quarantine in accommodation of their choice. Nationals, Permanent Residents (PRs) and long-term pass holders may serve their quarantine at their place of residence if suitable. 3. Category 3 – Unvaccinated travellers from countries in this category undergo 14-day quarantine at a dedicated facility. Vaccinated travellers may serve their 14-day quarantine at a suitable accommodation of their choice. 4. Category 4 – Travellers from all other countries required to quarantine for 14-days in a designated facility. Travellers from Bangladesh, India, Myanmar, Nepal, Pakistan, Sri Lanka are not allowed entry.  - 1 September 2021: Vaccinated Travel Lanes (VTL) created allowing international arrivals to undergo multiple PCR tests including a pre-departure PCR test within 48 hours of their scheduled departure for Singapore, an on-arrival PCR test upon arrival in Singapore and two additional PCR tests on Days 3 and 7 of their stay in Singapore.   Stabilisation Phase [27 September to 21 November 2021]   - 7 December 2021: Introduction of daily rapid testing regime for VTL travellers. All VTL travellers put on a daily rapid testing regime over 7 days on top of the PDT and on-arrival PCR tests. On Day 3 and 7, tests are done under supervision at a Test Centre.   Transition Phase [22 November 2021 to 25 April 2022]   - 16 February 2022: Fully vaccinated travellers arriving via VTL arrangements not subject to quarantine. A new “Restricted” travel category created for high risk countries warranting stricter measures - 22 February 2022: Quarantine standardized to 7 days across all countries. - 1 April 2022: Under the Vaccinated Travel Framework, all fully vaccinated travellers and non-fully vaccinated children permitted entry without quarantine.   Transition to COVID-19 Resilient Nation Phase [26 April 2022 to 12 February 2023]   - 29 August 2022: All non-fully vaccinated permitted entry without quarantine if they test negative within 2 days before departure.   Endemic Phase [ 13 February 2023 to Present]   - 13 February 2023: All travel measures lifted. |
| South Korea | - 3 January 2020:  Enhanced screening and quarantine required for all international arrivals from Wuhan, China after designation as a “quarantine inspection required area.” All arrivals are inspected by quarantine officers for symptoms, with symptomatic arrivals undergoing testing and potential quarantine. - 8 January 2020: COVID-19 designated a new infectious disease under the Act of Infectious Disease and Control,^^[[5]](#endnote-5)^^ and also a quarantinable infectious disease under the Quarantine Act.^^[[6]](#endnote-6)^^ - 28 January 2020: International arrivals who are Korean nationals, and display symptoms of COVID-19, to be hospitalized and undergo isolated treatment. - 28 January 2020: Enhanced screening and quarantine required for all international arrivals from Mainland China after designation as a “quarantine inspection required area.” - 30 January 2020: Around 250 staff of the Ministry of Health and Welfare, Ministry of National Defence and Korean National Police Agency are stationed at Incheon International Airport’s quarantine checkpoints. - 30 January 2020: Ministry of Health and Welfare announces US$4.5 million for quarantine and diagnostics as part of a US$17m Epidemic Prevention Budget. - 31 January 2020: First flight of repatriated nationals arrives with 368 passengers testing negative quarantined for 14 days. Two test positive passengers are isolated in hospital. - 1 February 2020: Second flight of 332 repatriated nationals arrives and placed in quarantine. - 4 February 2020:  International arrivals who are foreign nationals, and have travelled to Hubei Province within past 14 days, are prohibited from entering. Nationals, who have travelled to Hubei Province within the past 14 days, are required to undergo special immigration procedures including mandatory quarantine at home. - 12 February 2022: Enhanced screening and quarantine required for all international arrivals from mainland China, Hong Kong and Macau after designation as a “quarantine inspection required area.” - 12 February 2022: Third flight of 147 repatriated nationals arrives and is placed in quarantine. - 7 March 2020: Government announces GPS-based app to enforce self-quarantine measures. - 9 March 2020: Enhanced screening and quarantine required for all international arrivals from Japan after designation as a “quarantine inspection required area.” - 11 March 2020: Enhanced screening and quarantine required for all international arrivals from Iran and Italy after designation as a “quarantine inspection required area.” - 15 March 2020: : Enhanced screening and quarantine required for all international arrivals from France, Germany, Spain, UK and Netherlands, including transits through Dubai, Moscow and other countries after designation as a “quarantine inspection required area.” - 16 March 2020: Enhanced screening and quarantine required for all international arrivals from all European countries including transits through Dubai and other countries after designation as a “quarantine inspection required area.” - 19 March 2020: Enhanced screening and quarantine required for all international arrivals. - 22 March 2020:  All international arrivals planning long-term stay to self quarantine for 14 days. Foreign nationals actively monitored after quarantine. - 24 March 2020: Asymptomatic nationals must undergo quarantine at home for 14 days and be tested within 3 days of international arrival. Foreign nationals arriving from Europe must be tested and quarantined in a designated facility. Individuals who test negative, and plan long term stay, must quarantine at a domestic address for 14 days. - 25 March 2020:  Foreign nationals arriving from the USA must be tested and quarantined in a designated facility. Individuals who test negative, and plan long term stay, must quarantine at a domestic address for 14 days. - 1 April 2020:  All international arrivals must be tested and quarantine for 14 days. Foreign nationals arriving for short-term stay must quarantine at a designated facility at their expense. - 5 April 2020: International arrivals who do not comply with quarantine measures may face up to one year in prison or a US$8000 fine. - 11 May 2020: Foreign nationals who do not comply with mandatory self-quarantine measures upon arrival may be fined a maximum sixfold increase (US$46,000) - 31 May 2020: Government announces stronger quarantine measures for undocumented and foreign workers. - 5 May 2021:  International arrivals who are fully vaccinated in Korea, and undergo pre-departure and upon arrival PCR testing, are no longer required to quarantine. - 1 July 2021: International arrivals who are fully vaccinated abroad, visiting for specified purposes, and undergo pre-departure and upon arrival PCR testing, are no longer required to quarantine. 21 countries excluded from this exemption.^^[[7]](#endnote-7)^^ - 1 July 2021: International arrivals who are unvaccinated, but travelling for business or official reasons, and undergo pre-departure and upon arrival PCR testing, are no longer required to quarantine. - 1 November 2021: All international arrivals, regardless of nationality and vaccination status, and undergo pre-departure and upon arrival PCR testing, must be quarantined for 10 days. Previous exemptions for vaccinated travellers are suspended. - 6 February 2022: Government suspends use of GPS and daily checkup calls to low-risk individuals to monitor compliance with quarantine due to lack of capacity and high volume of Omicron infections. - 4 February 2022: All international arrivals, regardless of nationality and vaccination status, and undergo pre-departure and upon arrival PCR testing, must be quarantined for 7 days.^^[[8]](#endnote-8)^^ - 21 March 2022: : International arrivals who are fully vaccinated in Korea, regardless of nationality (except Pakistan, Uzbekistan, Ukraine and Myanmar), and undergo pre-departure and upon arrival PCR testing, are exempt from the 7-day quarantine requirement.^^[[9]](#endnote-9)^^ - 1 April 2022: International arrivals who are fully vaccinated abroad, regardless of nationality (except Pakistan, Uzbekistan, Ukraine and Myanmar), and undergo pre-departure and upon arrival PCR testing, are exempt from the 7-day quarantine requirement.^^[[10]](#endnote-10)^^ - 8 June 2022:  All international arrivals, regardless of nationality and vaccination status, and undergo pre-departure and upon arrival PCR testing, are no required to quarantine. |
| Taiwan | - 26 January 2020:  International arrivals who have travelled to Hubei Province must quarantine - 3 February 2020:  international arrivals via Mini-Three-Links (ships travelling between specific areas in Taiwan and specific areas in China) are required to quarantine - 6 February 2020:  International arrivals who have travelled in China, Hong Kong or Macau must quarantine - 19 March 2020:  Foreign nationals (except exempted categories) restricted from entering country. All international arrivals must quarantine for 14 days at their place of residence upon arrival.^^[[11]](#endnote-11)^^ - 6 November 2021: Introduction of strengthened quarantine measures for all international arrivals. All travelers are required to undergo a 14-day quarantine after arrival. All travelers are required to arrange their location of quarantine prior to arrival.^^[[12]](#endnote-12)^^ - 4 January 2022: International arrivals must test negative prior to departure and use the Quarantine System for Entry to pre-book a quarantine hotel or group quarantine facility prior to arrival.^^[[13]](#endnote-13)^^ - 7 March 2022: Mandatory quarantine period reduced from 14 days to 10 days followed by 7 days of self- monitoring. Non-nationals who are business travellers are permitted to enter the country. - 9 May 2022: Mandatory quarantine period reduced from 14 days to 7 days followed by self-health monitoring for all international arrivals. - 13 October 2022:  International arrivals are no longer required to quarantine but must undergo 7 days of self-monitoring.  If a traveller tests positive, they must be isolated. - 20 March 2023: Mild COVID-19 cases exempt from reporting and quarantine/isolation requirements. |

1. Australian Department of Home Affairs. *Inwards Travel Restrictions Operation Directive*, Canberra, 14 April 2022. <https://www.homeaffairs.gov.au/foi/files/2022/fa-220600418-document-released.PDF> [↑](#endnote-ref-1)
2. Te Tira Arai Uruta / NZ Royal Commission COVID-19 Lessons Learned. Whitiki Aotearoa: Lessons from COVID-19 to prepare Aotearoa New Zealand for a future pandemic Phase One, November 2024. <https://www.covid19lessons.royalcommission.nz/reports-lessons-learned/main-report/> [↑](#endnote-ref-2)
3. New Zealand. Ministry of Business, Innovation and Employment. *MIQ [Managed Isolation and Quarantine] timeline*. Auckland, 30 June 2023. <https://www.mbie.govt.nz/immigration-and-tourism/isolation-and-quarantine/managed-isolation-and-quarantine/about-miq/miq-timeline> [↑](#endnote-ref-3)
4. Singapore. Joint Press Statement by Ministry of Foreign Affairs and Ministry of Trade and Industry on the Singapore-China Fast Lane for Essential Travel. *Press Statement*, 3 June 2020. <https://www.mfa.gov.sg/Newsroom/Press-Statements-Transcripts-and-Photos/2020/06/20200603-SG-CHINA-Fast-Lane-Essential-Travel> [↑](#endnote-ref-4)
5. Korean Ministry of Health and Welfare. *Quarantinable Disease Risk Areas*. Centre for Disease Control and Prevention, Seoul, 8 January 2020. [↑](#endnote-ref-5)
6. Quarantine Management Team, COVID-19 National Emergency Response Center. Coronavirus Disease-19: Quarantine Framework for Travelers Entering Korea. *Osong Public Health and Research Perspectives* 2020; 11(3): 133-139. <https://ophrp.org/journal/view.php?doi=10.24171/j.phrp.2020.11.3.04> [↑](#endnote-ref-6)
7. Anon. South Korea: Authorities to introduce new COVID-19 restrictions scheme, ease measures nationwide from July 1 /update 62. *Crisis24*, 30 June 2021. <https://crisis24.garda.com/alerts/2021/06/south-korea-authorities-to-introduce-new-covid-19-restrictions-scheme-ease-measures-nationwide-from-july-1-update-62> [↑](#endnote-ref-7)
8. Consulate General of the Republic of Korea in San Francisco. *[COVID-19] Quarantine period will be shortened to 7 days for all travelers from overseas (starting from Feb 4th, 2022)*. Consular Notice, 28 January 2022. <https://overseas.mofa.go.kr/us-sanfrancisco-en/brd/m_22272/view.do?seq=45> [↑](#endnote-ref-8)
9. Korea Disease Control and Prevention Agency. KDCA Lifts Entry Restriction on Inbound Travellers from Abroad. Seoul, 31 March 2022. <https://kdca.go.kr/board/board.es?mid=a30402000000&bid=0030> [↑](#endnote-ref-9)
10. Anon. South Korea: Officials to ease quarantine measures for fully vaccinated travelers from March 21 /update 1. *Crisis 24*, 14 March 2022.

    <https://crisis24.garda.com/alerts/2022/03/south-korea-officials-to-ease-quarantine-measures-for-fully-vaccinated-travelers-from-march-21-update-1> [↑](#endnote-ref-10)
11. Taiwan Ministry of Foreign Affairs. Taiwan to bar foreign nationals from entering the country starting March 19 in response to the continued spread of COVID-19. *Press Release*, Bureau of Consular Affairs, 18 March 2020. <https://en.mofa.gov.tw/News_Content.aspx?n=1EADDCFD4C6EC567&s=1816E365AF525FDD> [↑](#endnote-ref-11)
12. Taiwan Ministry of the Interior. Restrictions on Entering Taiwan - ¼ - Taiwan Nationals, Foreign Nationals. Taipei, 6 November 2021. <https://www.roc-taiwan.org/uploads/sites/32/2021/12/1101106-restrictions-on-entering-taiwan-1.pdf> [↑](#endnote-ref-12)
13. Taiwan Centre for Disease Control. Entry and Quarantine Measures for COVID-19 Prevention FAQs concerning COVID-19 RT-PCR test reports within two days of boarding. Taipei, January 2022. <https://www.cdc.gov.tw/Uploads/dfbbc2b5-34d0-458a-b1e4-6477e551c232.pdf> [↑](#endnote-ref-13)
