# Supplementary material for: The use of quarantine as an international travel measure during the COVID-19 pandemic: A comparative analysis of implementation and equity impacts in five “exemplar” countries
Source: PLOS Glob Public Health. 2025 Nov 14;5(11):e0005457. doi: 10.1371/journal.pgph.0005457 (PMC12617841; doi:10.1371/journal.pgph.0005457)
Supplement: S3 Annex — (DOCX) [file pgph.0005457.s003.docx]

**S3 Annex: Length of quarantine period during the COVID-19 pandemic in the five countries**

| Australia | - minimum 14 days (from 29 January 2020) - 3 days (from 27 November 2021 by some states/territories for international arrivals from low-risk countries) - 0 days (from 15 December 2021 by some states/territories for international arrivals who are vaccinated and test negative) - 7 days (from 21 February 2022 for unvaccinated international arrivals) - 0 days (from 1 June 2022 for all international arrivals to all states/territories regardless of nationality or vaccination status) |
| --- | --- |
| Aotearoa New Zealand | - minimum of 14 days (from 16 March 2020) - 7 days from 28 October 2021 (with self-quarantine pending test result) - 10 days (from 21 December 2021 in response to Omicron variant) - 0 days (from 3 May 2022) |
| Singapore | - 14 days (from 21 March 2020 to 21 August 2020) - 7 days (from 1 September 2020 for low-risk countries) - 0 days for travel between Hong Kong and Singapore with 2 tests (planned for 22 November 2020 but delayed indefinitely on 1 December 2020) - 0 days for essential business travellers based in Singapore with a tightly controlled itinerary, on-arrival COVID-19 PCR test and additional PCR tests on Day 3, 7 and 14 of their return and self-isolate at designated facilities for up to 14 days (from June 2020 to 1 Sep 2021) - 14 + 7 days for citizens/residents arriving from high-risk countries (from 8 May 2021 till 24 June 2021) - 14 days for all travellers (from 25 June 2021) - 7 or 14 days based on risk category assigned to country, from Cat 1 to Cat 4 (from 19 Aug 2021) - 0 days for vaccinated travellers from selected countries through the Vaccinated Travel Lanes program with negative COVID-19 PCR 48 hours prior to travel and on-arrival PCR test (from 19 October 2021 to 23 December 2021) - 7 days for all countries (from 22 February 2022) - 0 days and no post arrival testing for vaccinated travellers from all countries; 7 days for unvaccinated or not fully vaccinated travellers (from 1 April 2022) - 0 days for unvaccinated or not fully vaccinated travellers with pre-departure PCR test (29 August 2022) - 0 days for all international arrivals (from 1 April 2023) |
| South Korea | - 14 days (from 22 March 2020) - 10 days (from 1 November 2021) - 7 days (from 4 February 2022) - 0 days for all international (from 8 June 2022) |
| Taiwan | - 14 days (from 26 January 2020) - 10 days (from 7 March 2022) - 7 days (from 9 May 2022) - 3 days (from June 15, 2022) - 0 days (from 13 October 2022 when quarantine requirement lifted) |
