# Supplementary material for: The use of quarantine as an international travel measure during the COVID-19 pandemic: A comparative analysis of implementation and equity impacts in five “exemplar” countries
Source: PLOS Glob Public Health. 2025 Nov 14;5(11):e0005457. doi: 10.1371/journal.pgph.0005457 (PMC12617841; doi:10.1371/journal.pgph.0005457)
Supplement: S4 Annex — (DOCX) [file pgph.0005457.s004.docx]

**S4 Annex:** **Persons/populations subject to travel-related quarantine in the five countries**

| Australia | - Initially risk assessed by source country for travel - All international arrivals from 16 March 2020 - From November 2021 there was some variation based on vaccination status, where vaccinated nationals and residents could enter quarantine free assuming negative test before flying and upon arrival - There was a brief quarantine-exempt ‘travel bubble’ between Australia and New Zealand 20 April-23 July 2021 - Some international arrivals were exempted from supervised (hotel) quarantine: foreign diplomats and Australian government officials, travelers with medical requirements that could not be met in designated facilities, air crew, oil/gas and maritime workers. Some exceptions were also made on ‘compassionate’ grounds, including high profile people from the entertainment industry. |
| --- | --- |
| New Zealand | - All international arrivals from 19 April 2020 - Some international arrivals exempted from supervised (hotel) quarantine (e.g., diplomats, aircrew, and people under strict criteria (i.e., quarantine at home) - Some travellers from low-risk Pacific Islands exempted from MIQ during parts of 2020-2021. All arrivals from low-risk Pacific Islands exempted from 8 November 2021 - There was a brief quarantine-exempt ‘travel bubble’ between Australia and New Zealand from 20 April-23 July 2021 |
| Singapore^^[[1]](#endnote-1)^^ | - All international arrivals required to quarantine from 21 March 2020 - Short-term travellers (non-citizens/residents) not allowed to enter Singapore from 23 March 2020 - The specific quarantine requirements (i.e., where, how long, what testing) for individual travellers were dependent on the assessed risk category of source country, travel history, citizenship/residency status, and vaccination status. - Multiple exemptions were introduced to allow essential travel to take place:   - Business Travel Pass (BTP) introduced in June 2020 to facilitate business travel for senior executives based in Singapore with regional or international responsibilities. BTP travellers were required to undergo PCR testing upon arrival, self-quarantine until a negative test result (positive test result requires continued quarantine), and a strictly controlled itinerary in lieu of quarantine.   - “Fast Lane” arrangement introduced in June 2020 between Singapore and China to facilitate business travel and official purposes   - “Reciprocal Green Lane (RGL)” scheme introduced from September 2020 to allow travel between Singapore and low-risk countries.   - “Vaccinated Travel Lanes” introduced for fully vaccinated travellers entering Singapore from 8 September 2021 - Travellers entering Singapore under these schemes subjected to strict testing regimes instead of serving a SHN/quarantine |
| South Korea | - Quarantine measures based on travel history. All international arrivals from high risk countries required to quarantine from 3 January-30 March 2020. Number of source countries assessed as high risk increases over time. - All international arrivals required to quarantine from 1 April 2020. - Certification of Quarantine Exemption issued by Korean embassies introduced for eligible travellers based on purpose of visit. - Critical Business: A-1(Diplomacy), A-2(Official Business), or A-3 visa holders, SOFA Stamp holders(exemption, but they need a PCR testing in the U.S Army units) - Academic, public purpose: national event. (Only companies or organizations can apply to get the certification. Individuals are not eligible to apply.) - Humanitarian purpose: Attending a funeral (spouse, siblings of spouse, parents or child) - Korea-Singapore Vaccinated Travel Lane created whereby travellers do not need Certification of Quarantine Exemption. Travelers must be fully vaccinated in Korea or Singapore from 15 November 2021^^[[2]](#endnote-2)^^ |
| Taiwan | - Quarantine measures applied dependent on travel history from 26 January 2020. - All international arrivals required to quarantine from 19 March 2020. - Exemptions based on "air bridges," "air corridors," "travel bubbles" for specification diplomacy or business purposes approved by the government are exempted from the quarantine regulation. - Airline crew members subjected to special regulation: - From 19 March 2020: 14 days for all crew members - From 27 March 2020: 3 days for Cargo crew members, 5 days for passenger aircraft crew members - From 1 January 2021: 7 days for all crew members - From 12 March 2021: 5 days for all crew members - From 15 April 2021: 3 days for all crew members - From 15 June 2022: 0 days for fully vaccinated crew members |

1. Singapore. [Updated] Facilitating the resumption of travel with the Vaccinated Travel Framework. 24 August 2022. [https://www.gov.sg/article/facilitating-the-resumption-of-travel-using-the-vaccinated-travel-framework#:~:text=Since%20its%20launch%20on%201,or%20application%20for%20entry%20 approvals](https://www.gov.sg/article/facilitating-the-resumption-of-travel-using-the-vaccinated-travel-framework#:~:text=Since%20its%20launch%20on%201,or%20applying%20for%20entry%20approvals) [↑](#endnote-ref-1)
2. Cheng I. Singapore, South Korea to launch vaccinated travel lanes on Nov 15. *CNA*, 8 October 2021.

   <https://www.channelnewsasia.com/singapore/covid-19-singapore-south-korea-vaccinated-travel-lane-vtl-nov-15-2230431> [↑](#endnote-ref-2)
