# Supplementary material for: The use of quarantine as an international travel measure during the COVID-19 pandemic: A comparative analysis of implementation and equity impacts in five “exemplar” countries
Source: PLOS Glob Public Health. 2025 Nov 14;5(11):e0005457. doi: 10.1371/journal.pgph.0005457 (PMC12617841; doi:10.1371/journal.pgph.0005457)
Supplement: S5 Annex — (DOCX) [file pgph.0005457.s005.docx]

**S5 Annex: Location of quarantine by international travellers in the five countries**

| Australia | - International arrivals (nationals and residents) permitted to quarantine at home until 27 March 2020. - Mandatory quarantine in designated facilities introduced on 27 March 2020 for all international arrivals. This requirement remained in place until November 2021. - Hotels were used as designated facilities throughout the country. - A miners camp in the Northern Territory was repurposed and used to quarantine arrivals on repatriation flights. - Three Centres for National Resilience in Melbourne, Perth and Brisbane (2000-bed capacity) were completed by March 2022 to supplement existing quarantine arrangements and provide emergency accommodation.^^[[1]](#endnote-1)^^ |
| --- | --- |
| Aotearoa New Zealand | - Home until April 27 2020, then designated facilities (all repurposed hotels). - From late 2021 some eligible travellers were permitted to combine hotel (7 days) with home (3 days) |
| Singapore | - When mandatory quarantine was announced on 21 March 2020, travellers were first required to make their own accommodation arrangements, either at a hotel or their place of residence. Singapore Citizens and Permanent Residents were allowed to quarantine in their residences if they travelled from low-risk countries. - From 25 Mar 2020, dedicated facilities (mainly hotels converted into quarantine sites) were set up for travellers to serve their SHN. - Dedicated SHN facilities were the **default sites** to serve quarantine. However, based on the risk status of different countries, travellers from low-risk countries were allowed to serve their SHN at places of residence and were given the option to either stay at dedicated SHN facilities or their own places of residence. Travellers from high-risk countries, however, were required to serve their SHN at these dedicated SHN  facilities. - Work Permit holders (mainly migrant workers employed in Construction, Marine Shipyard and Process sectors and staying in dormitories) were required to serve their SHN at dedicated SHN facilities for 14 days, followed by 7 days at Migrant Worker Onboarding Centres (MWOCs). From 15 Mar 2021, this process was streamlined with the setting up of more onboarding centres. Under this process, migrant workers arriving in Singapore will spend about 3 to 4 days at dedicated SHN facilities (mainly hotels) to wait for the results of their serology and polymerase chain reaction (PCR) Covid-19 tests. After that, workers will be moved to onboarding centres, within five quick-build dormitories in Punggol, Eunos, Choa Chu Kang and Tengah, to serve the rest of their quarantine period.^^[[2]](#endnote-2)^^ |
| South Korea | - For test-positive (Post-arrival Test) - hospital or (designated)residential treatment center - For test-negative - Korean and long-term stay foreigners: home(they have to install Self-Quarantine Safety Protection App, and self-report about their health condition for 14 days) - short-term stay foreigners: designated facility (Exception: allowed to stay at home in cases who were related to Korean or long-term resident foreigners as spouse, parent, child, sibling, and uncles/aunts only). |
| Taiwan | - Starting 26 January 2020, international arrivals were required to quarantine at home or a government quarantine facility. - From March 2020-March 2022, travellers were given a choice to quarantine at home, government quarantine facility, or designated hotel. |

1. Australia Department of Finance. *Centres for National Resilience*. Canberra, 17 November 2022. <https://www.finance.gov.au/government/property-and-construction/centres-national-resilience> [↑](#endnote-ref-1)
2. Sen NJ. Newly arrived migrant workers in 3 sectors to have shorter SHN stays in hotels under onboarding pilot. *Today*, 3 March 2021. <https://www.todayonline.com/singapore/newly-arrived-migrant-workers-spend-fewer-days-hotels-shn-under-new-onboarding-pilot> [↑](#endnote-ref-2)
