# Supplementary material for: The use of quarantine as an international travel measure during the COVID-19 pandemic: A comparative analysis of implementation and equity impacts in five “exemplar” countries
Source: PLOS Glob Public Health. 2025 Nov 14;5(11):e0005457. doi: 10.1371/journal.pgph.0005457 (PMC12617841; doi:10.1371/journal.pgph.0005457)
Supplement: S6 Annex — (DOCX) [file pgph.0005457.s006.docx]

**S6 Annex: How much and who paid for quarantine in the five countries**

| Australia  US$1=  A$1.392 | How much: From July 2020, around A$3000 (US$2155) for the first adult and A$1000 (US$1392) per subsequent adult for a 14-day period.  Cost per child varied by state/territory. Included accommodation, food, transport, testing and accommodation.  Who paid:  State/federal governments paid all costs until July 2020, when the above charges were introduced. Travellers could apply for fee reductions or waivers on financial hardship grounds after bills for quarantine were issued, which was some time after their stay was completed. In practice, many hotel quarantine bills remain unpaid. . |
| --- | --- |
| Aotearoa New Zealand  US$1=  NZ$1.478 | How much: Cost depended on length of stay and citizenship/visa status. Nationals cost NZ$3100 (US$2,097) for the first adult plus NZ$950 (US$643) per additional adult and NZ$475 (US$321) per additional child for a 14-day period. Non-citizens/permanent residents cost $5250 (US$3552) for the first adult plus NZ$2990 (US$2023) per additional adult and NZ$1690 (US$1143) per additional child. Included accommodation, food, transport, testing and accommodation.  Who paid: “On 11 August 2020, the MIQ fees regime came into force to support the financial sustainability of the MIQ system. New Zealanders who left New Zealand before the regime came into force who enter for less than 90 days [later 180 days], or who leave New Zealand after the regime came into force are now charged (although full or partial waivers are available). Temporary visa holders currently have to pay, unless they left New Zealand on or before 19 March 2020, and were ordinarily resident in New Zealand as of 19 March 2020. All critical workers entering New Zealand are liable for charges at a higher rate than New Zealand citizens and other visa holders.”^^[[1]](#endnote-1)^^ |
| Singapore  US$1=  S$1.362 | How much:  Cost depended on length of stay and standard of accommodation. Minimum of S$1,000 (US$734) per person for 7-day period; S$2,000 (US$1468) per person for 14-day period; and S$3,000 (US$2,203) per person for a 21-day period. Quarantine in a luxury hotel could cost up to S$20,000 (US$14,684) for a 14-day period. Cost of COVID-19 testing (S$200/US$147 per test) was included.  Who paid: From introduction of mandatory quarantine on 21 March 2020 to 17 June 2020, the government paid the costs of nationals and permanent residents staying at dedicated facilities. This excluded nationals/PRs who left Singapore after the travel advisory not to travel internationally issued 27 March 2020. From 18 June 2020, all inbound travellers paid the cost of staying at dedicated facilities and required testing.^^[[2]](#endnote-2)^^  On 5 January 2021, the Ministry of Manpower issued a directive that the cost of quarantine in a hotel and then designated facility, for newly arrived migrant workers who reside in dormitories (employed in the Construction, Marine and Process sectors), should be covered by employers. Despite this advisory, some employers passed on the cost of serving quarantine and testing to migrant workers entering Singapore. The migrant worker advocacy NGO Transient Workers Count Too (TWC2) reported that while “employers must bear the cost in full and are not allowed to pass on any cost to the employees,” some employers were requiring employees to pay.^^[[3]](#endnote-3)^^ |
| South Korea  US$1=1,162 Won | How much: Based on length of stay. Food, accommodation and transportation costs are ₩100,000-150,000 per day (US$86-$129). Approximate cost of ₩1,400,000-2,100,000 (US$1,205-$1,807) for 14-day period. Testing costs included.  Who Paid: Travellers paid the full amount for quarantine upon arrival. If travellers tested positive, and required isolation and/or treatment, government paid these costs. Cost sharing for test-positive travellers depended on nationality. For nationals, local and central governments (KDCA) paid 50% of costs each. For non-nationals, central government paid 100% of costs. |
| Taiwan  US$1=  TW$28.696 | How much: Cost (including food, testing and accommodation but not transport) depended on length of stay, location and standard of accommodation.^^[[4]](#endnote-4)^^ Government facilities cost US$64 per person per day. Hotels varied, depending on the price levels of different counties and cities, from US$32 to US$510 per person per day. Higher costs were charged depending on such factors as room type, size, balcony, bathtub, and external windows.  Who paid: Travellers paid costs. However, the central government provided a daily subsidy of US$32 during the quarantine period, while some local governments offered an additional subsidy of US$16 per day. Residents could receive a maximum subsidy ranging from US$669-$765.  Whether quarantined after entering Taiwan or isolated after testing positive, incoming travelers must initially pay for quarantine/isolation facilities themselves. They can later apply to the government for a subsidy for quarantine or isolation expenses. However, only Taiwanese citizens and foreign residents with resident certificates are eligible for this subsidy. In other words, incoming travelers who are not Taiwanese citizens and do not have resident certificates must bear the costs of quarantine/isolation facilities and treatment themselves. |

Note: Currency exchange rate used are average between January 2020-December 2021. See <https://www.irs.gov/individuals/international-taxpayers/yearly-average-currency-exchange-rates>

1. Ministry of Business, Innovation and Employment. *Managed Isolation and Quarantine charges for temporary entry class visa holders and extension of charging for MIQ for returning New Zealanders*. Government of New Zealand, Auckland, 21 June 2021. <https://www.mbie.govt.nz/dmsdocument/15131-supplementary-analysis-report-amendments-to-the-covid-19-public-health-response-managed-isolation-and-quarantine-charges-regulations-2020-proactiverelease-pdf> [↑](#endnote-ref-1)
2. <https://www.mfa.gov.sg/Overseas-Mission/Mumbai/Announcements/Travellers-to-bear-costs-of-COVID-19-tests-and-stay-at-Dedicated-SHN-Facilities> [↑](#endnote-ref-2)
3. <https://twc2.org.sg/2021/03/12/employers-claw-back-cost-of-stay-home-period-from-arriving-workers/> [↑](#endnote-ref-3)
4. Asiayo. *Taiwan Quarantine Hotel*. n.d. <https://asiayo.com/event/english-home-isolation.html> [↑](#endnote-ref-4)
