# Supplementary material for: The use of quarantine as an international travel measure during the COVID-19 pandemic: A comparative analysis of implementation and equity impacts in five “exemplar” countries
Source: PLOS Glob Public Health. 2025 Nov 14;5(11):e0005457. doi: 10.1371/journal.pgph.0005457 (PMC12617841; doi:10.1371/journal.pgph.0005457)
Supplement: S7 Annex — (DOCX) [file pgph.0005457.s007.docx]

**S7 Annex: How quarantine facilities were accessed in the five countries**

| Australia | All international arrivals were required to quarantine for 14-days in designated facilities. Given limited rooms available in quarantine hotels, the volume of international arrivals was reduced by restricting the number of inbound arrivals permitted to land. The total number of allowed arrivals was managed on an ongoing basis by the Commonwealth Cabinet, and arrival caps were communicated to airlines. The airlines then controlled access to seats on available flights which often depended on ability to pay (i.e., business class tickets). International arrivals were assigned accommodation at a designated facility (hotel) upon arrival, except for arrivals into the Northern Territory who all stayed at the same repurposed mining camp. Assignments were mostly random although this varied by the location of the point of entry. Some efforts were made to assign larger rooms or suites with kitchens to groups of travellers (e.g. families). |
| --- | --- |
| AotearoaNew Zealand | From July 2020, at the government’s request, Air New Zealand reduced or ceased flight arrivals to allow management of demand for quarantine. During this period, international arrivals were assigned accommodation at a designated facility (hotel) upon arrival. From October 2020 travellers were required to use an on-line booking system to reserve a room before booking their flight, and were legally prevented from flying without a confirmed MIQ place. The system was used to manage available supply with demand, although a long waitlist for quarantine rooms resulted. To manage this, an online lottery for places was established and reset each week. A priority system was created to allocate places in cases of emergency. |
| Singapore  [Updated] | International arrivals who were required to quarantine in designated facilities were randomly allocated places upon arrival. Travellers could also pre-book accommodation from a list of approved quarantine hotels.  Migrant workers served their SHN at dedicated facilities (mainly hotels) for 21 days initially. From 15 March 2021, migrant workers spent 3-4 days at dedicated facilities (mainly hotels) and then moved to onboarding centres (five quick-build dormitories in Punggol, Eunos, Choa Chu Kang and Tengah) for the remaining quarantine period. Prior to arrival, employers pre-selected and booked the facility/hotel for their workers via a Ministry of Manpower online portal. |
| South Korea | - Assigned upon arrival. Mostly randomly but some facilities were designated for foreign nationals only. - Access to quarantine site - stay-at-home: own car, COVID-19 free taxi   “We strongly recommend using your own vehicle for returning home, but when it is difficult to access their car, the government provides designated buses and trains(separated train compartment) for arrivals.”   - the fare is the same as the regular fare for transportation - designated facility: take a police-escorted bus at the airport to minimize contact with other incoming travelers |
| Taiwan | International arrivals quarantining either at home (nationals) or in government facilities. Foreign nationals needed to book in advance to rent a room but the specific facility was assigned upon arrival. Travelers quarantining in designated hotels could make reservations through online booking websites, instant messaging software such as Line, or by phone. |
