# Supplementary material for: The use of quarantine as an international travel measure during the COVID-19 pandemic: A comparative analysis of implementation and equity impacts in five “exemplar” countries
Source: PLOS Glob Public Health. 2025 Nov 14;5(11):e0005457. doi: 10.1371/journal.pgph.0005457 (PMC12617841; doi:10.1371/journal.pgph.0005457)
Supplement: S8 Annex — (DOCX) [file pgph.0005457.s008.docx]

**S8 Annex: Conditions of quarantine in designated facilities in the five countries**

| Australia | **Food:** 3 meals per day with some variation by facility e.g., 3 times per day or one day supply of food at once. Food reported as low quality although this is recognized as subjective. Some facilities offered ‘special menus’ at additional cost or catered to special dietary needs. Some provided food appropriate for children. Some allowed unlimited deliveries using food apps, others restricted them.  **Amenities and facilities:** Varied by location. Standard hotel rooms did not have opening windows or additional facilities. Serviced apartments were more spacious with kitchen and/or laundry facilities. Outdoor space depended on the facility and the capacity to manage access. If available, 30-minute slots could be booked several times per week. The repurposed mining camp had limited facilities but provided access to verandah and outdoor spaces.  **Healthcare:** People needing healthcare could use telehealth and be transferred to ‘Special Health Accommodation’ if necessary (i.e. hotel or similar run by state health authorities rather than the police or defence force).  **Special needs**: People with selected special needs, who could not be safely accommodated in designated facilities or Special Health Accommodation, could apply to quarantine at home. Exemptions were not easily granted.  **Priority populations:** Varied by location. All travellers were assumed to possess a mobile phone or other electronic device. This reportedly left some older adults without access to communications or entertainment.  **Cultural accommodations:** Varied by location. Some were more able to cater to different dietary needs than others. |
| --- | --- |
| New Zealand | **Food:** 3 meals per day. Food reported as low quality although this is recognized as subjective. Some but not all facilities were able to cater well to special dietary needs.  **Amenities and facilities**: Outdoor space available in some facilities but dependent on traveller’s test status and facility’s capacity to manage access. Improvements to access to fresh air and outdoor space were made later in 2020. This included facilitating physically distanced visits from family and loved ones at MIQ perimeter fences where possible.^^[[1]](#endnote-1)^^  **Healthcare:** Telehealth and other health services were tailored to specific populations and language groups. Registered nurses were on site. Proactive support reportedly depended on the facility.^^[[2]](#endnote-2)^^  **Special needs**: MIQ staff were not made aware of special needs before travellers arrived. An ombudsman report said detainees’ special needs were, on the whole, met.  **Priority populations:** Varied by location, some were able to cater more appropriately for children and people with physical disabilities.  **Cultural accommodations:** Interpreters were available, catering to cultural dietary needs varied by location. |
| Singapore | Quarantine facilities were mainly hotels converted into SHN sites for travellers and quality depended on the hotel (5-star hotels had better rooms). However, travellers were confined to their rooms and meals were all catered for them with limited/no choice.  **Food:** 3 meals per day. Food reported as low quality although this is recognized as subjective. Travellers were not allowed to choose their food options other than indicating dietary restrictions or allergies. The option to order food through food delivery apps was available although expensive.  **Amenities and facilities**: Varied by location depending on hotel standard. Hotel rooms were typically single rooms with ensuite bathrooms and internet access. No outdoor spaces provided. Some rooms had balcony access although these were mainly at more expensive hotels.  **Healthcare:** Telemedicine services were available to travellers serving quarantine if they felt unwell. Medications given by telemedicine providers were delivered to the hotel rooms of travellers. Travellers who tested positive in quarantine but felt generally well, had to continue self-isolation in their hotel rooms until they tested negative. For travellers who were unwell or needed specialized medical attention, hotel staff arranged for them to be transported through dedicated transfer to the National Centre for Infectious Diseases or to other public hospitals for treatment. Travellers were responsible for all costs associated with recovery, testing and treatment, including any necessary extended stay in hotels or care facilities.  **Special needs:** No provisions found.  **Priority populations:** Families with children were allowed to stay in the same or adjoining rooms depending on availability. Young children were allowed to quarantine with their parents.  **Cultural accommodations:** The general quality of food was mediocre and not culturally suitable for travellers of different nationalities. Dietary requirements, such as halal food for Muslims, as well as any special diets due to allergies/medical reasons were catered for, with no other provisions made.For instance, migrant workers from the main sending countries, Bangladesh and India, did not find the food options provided for them palatable/ tailored to their taste preferences (e.g. most reported it was bland). |
| South Korea | **Food:** 3 meals plus snacks per day.  **Amenities and facilities**: Single bedrooms, internet access, no outdoor space (not permitted to leave designated space)  **Healthcare:** On-site medical staff monitored the health status of quarantined individuals including mental health needs (initial counseling provided if required). Individuals referred to specialized agencies or national trauma centers if treatment was needed.  **Special needs:** no data  **Priority populations:** One designated caregiver permitted to stay with vulnerable populations, such as infants, older adults and people with disabilities, to ensure safety.  **Cultural accommodations**: Special food for religious reasons was provided. A prayer room was provided upon request in facilities for foreign nationals. |
| Taiwan | **Food:** Varied by location and individual preferences. Reported complaints in the media about some food being unpalatable or spoiled.  **Amenities and facilitie**s: In government facilities, rooms are equipped with internet, television, and basic room related amenities, along with thermometers and bathroom supplies. Cleaning products are provided, and residents are expected to wash their clothes inside their rooms. The staff at the quarantine facilities, including the health team, security team, and logistics team, offer services to those in quarantine during their stay. In quarantine hotels, rooms came with basic amenities such as private bathroom, air conditioning; telephone, internet, and television, and hygiene supplies. Higher-priced rooms included access to fitness equipment, video gaming devices, bathtubs, balconies, etc. Some lower-priced rooms may have no windows.  **Healthcare:** Strong focus on infection control including separate pathways, temperature monitoring at entrances and exits, and cleaning supplies. Government quarantine facilities are staffed with medical personnel. Those staying in quarantine hotels can request assistance for health needs through a government-established helpline or an official communication software chatbot. Individuals quarantining at home can reach out for help by calling the helpline, using the official chatbot, or contacting the village representative's office.  **Special needs:** For humanitarian considerations, non-symptomatic individuals in quarantine can apply to local health authorities for permission to leave for mourning or visiting due to emergencies such as a family member's death or serious illness. Pets could not be accommodated in government quarantine facilities or quarantine hotels, but were placed in temporary pet lodging facilities at the owner's expense. Government quarantine facilities and quarantine hotels do not offer a shopping service for goods. Residents can order delivery meals on their own or have family members deliver items, which can then be passed on to the residents by the facility staff. However, the transfer of cigarettes, alcoholic beverages, high-energy-consuming appliances, and other hazardous items (such as lighters, knives) was not permitted.  **Priority populations:** Individuals in quarantine who meet the following conditions can apply for a caregiver to accompany them during quarantine:   - Children and adolescents (under 18 years old). - Individuals over 65 years old who are unable to take care of themselves. - Persons with disabilities. - Individuals with physical or mental impairments. - Those with serious illnesses. Pregnant individuals deemed by a physician to be in need of care and unable to take care of themselves. - Individuals unable to take care of themselves due to injury or illness. - Other cases approved upon evaluation by the local government.   **Cultural accommodations**: no data |

1. Office of the Ombudsman Tari o te Kaitiaki Mana Tangata. Thematic Report on Inspections of Managed Isolation and Quarantine Facilities under the Crimes of Torture Act 1989 [Internet]. 2021 Aug. Available from: <https://www.ombudsman.parliament.nz/sites/default/files/2021-08/Thematic%20report%20on%20inspections%20of%20Managed%20Isolation%20and%20Quarantine%20Facilities%20under%20the%20Crimes%20of%20Torture%20Act%201989.pdf> [↑](#endnote-ref-1)
2. <https://www.stuff.co.nz/national/health/coronavirus/124648423/from-day-0-to-day-14-what-its-like-inside-a-covid19-managed-isolation-facility> [↑](#endnote-ref-2)
