# Supplementary material for: The use of quarantine as an international travel measure during the COVID-19 pandemic: A comparative analysis of implementation and equity impacts in five “exemplar” countries
Source: PLOS Glob Public Health. 2025 Nov 14;5(11):e0005457. doi: 10.1371/journal.pgph.0005457 (PMC12617841; doi:10.1371/journal.pgph.0005457)
Supplement: Attachment — Submitted filename: PLoSGlobalHealth_quarantine-responsetoreviewers.docx [file pgph.0005457.s010.docx]

**The use of quarantine as an international travel measure during the COVID-19 pandemic: A comparative analysis of implementation and equity impacts in five “exemplar” countries**

**Response to Reviewers**

Reviewer 1

1. *This is an insightful article and a much-appreciated review of quarantine practices during the pandemic.*

We thank the reviewer for this supportive comment.

2. *Would suggest you consider the criteria listed from SARS in Toronto -* [*https://pmc.ncbi.nlm.nih.gov/articles/PMC2094974/*](https://pmc.ncbi.nlm.nih.gov/articles/PMC2094974/)

We appreciate this suggestion to consider using the following three criteria for quarantine to be a useful measure of disease control:

- first, people likely to be incubating the infection must be efficiently and effectively identified;
- second, those people must comply with the conditions of quarantine; and
- third, the infectious disease in question must be transmissible in its presymptomatic or early symptomatic stages.

While helpful for assessing whether to use quarantine for disease control, our paper is not an assessment of the appropriateness or effectiveness of the use of quarantine to achieve public health goals. The five countries analyzed were selected because they are deemed exemplary in this respect. Our paper seeks to compare how quarantine was used in each country to identify similarities and differences in practices. This comparison, in turn, can offer better understanding of the secondary social or economic impacts of quarantine including equity considerations. We make this purpose clearer in our revised Abstract, Introduction and Discussion, also adding a citation to the reviewer’s suggested paper.

3. *Would suggest you lightly touch on what type of government/political system is in place within each country.*

We have added the question “What type of political system does each country have?” to Table 2 and brief information on the political system for each country in Table 3.

4. *What about in-country travel across territories? Thinking of NSW vs QLD, etc. Realizing this is focused on intl travel, but might be worth mentioning.*

Of the five countries, only Australia sought to manage public health risks from domestic travel. While travel restrictions, testing and vaccination were used for this purpose, quarantine was not one of the measures used. As the paper is focused on the comparative use of quarantine for international travel in five countries, we have decided that mention of Australia’s use of other types of travel measures for domestic use is not directly relevant.

5. *Would also consider noting that the reliance on such significant quarantine measures often impacted other response - such as the rollout of vaccines (thinking Aus), which then allowed for significant outbreaks (e.g. Sydney cruise ship) and false sense of security.*

This is indeed true and we have added a brief point about the importance of wider, often unanticipated effects, of specific policy measures during COVID-19 in the Discussion. However, the paper is not an assessment of the effectiveness of quarantine in achieving public health outcomes in each country. We are also seeking comparative findings and examples of similar knock on effects are not available from the other four countries.

Reviewer 2

1. *The paper has an excellent start with the information on international travel measures (ITM) and its role on achieving public health goals during pandemic. However, the introductory paragraphs could be rearranged under single heading, Introduction, to establish logical and improve readability as: Concept of ITM, quarantine (page 16-17, conditions of quarantine), and isolation (Page 4, 1st and 2nd para.), equity lens (Page 7, 3rd. para.), literature summary (Table 1), evidence gap (Page 3, 2nd. and 3rd para.), and objectives. It is suggested to highlight the existing evidence with appropriate paraphrasing and citation rather than mentioning it with “….” (for instance, Page 5, line 7-13).*

Thank you for these suggestions. For improved flow and readability, we have substantially revised the Introduction as suggested. This includes reducing and integrating the literature review presented in the Background into the Introduction. We have also decided to eliminate Table 1, retaining only the key point about the need to better understand context within which quarantine is used as an ITM, as it did not add substantively to the paper. This also helped reduce the length of the paper.

2. *Additionally, the last paragraph of the introduction section (Page 4) might be more appropriate in the approach and methods section, as it delves into methodological details. It is suggested to differentiate primary and secondary data mentioned on Page 6, line 1, followed by case study selection, frameworks, data collection tools and coding of quarantine practices. I am curious about the 0-1 scale used for quarantine policy (Page 7, line 1-3). Moreover, the paragraphs explaining equity lens, on equity framework (Page 7-8) is insightful. However, considering the section where it is placed, it can be made more concise, limiting to the type of justice and relevant questions. A table with detailed information can be added on supplementary file for reference.*

We have rewritten the Introduction as described in comment #1. We have decided not to move the final paragraph of the Introduction to the Approach and Methods section because this paragraph is briefly providing an outline of the paper. However, we have also rewritten the Approach and Methods section as advised. The material has been resequenced and more details added on the data sources. We also moved the final section of the Background to the Approach and Methods section.

3. *The depth of information provided in the results section, under the components (legal mandate, timing, duration, population, location, cost, access, conditions) of the heuristic framework for comparative analysis of quarantine implementation and equity framework is commendable. However, as a reader, I felt it would more appropriate to discuss the first paragraph (page 8-9) in Introduction/Discussion section. Moreover, a few sentences seemed to lack the needful information as well. [For instance, page 8, last para., line 2- included in which implementation framework? Page 10, 1st. para, line 1- which 4 countries embedded quarantine within broader legislation? And line 2- which three countries made temporary expansion ?] Page 10, last para., line 7: can we mention the word “perhaps” in the result section ?*
4. *Further, it is suggested to insert footnotes for terms like travel bubbles, travel corridors (page 11, 2nd para).*

We have rephrased and added definitions in the text.

5. *Further, I felt there are few paragraphs in the results section which has better scope in the discussion section of the manuscript. For instance, page 17 last para. Similarly, the findings on page 20, 2nd para is an interesting one but this section requires logical flow and connection between the paragraphs. Else, it can be shifted in discussion section as well.*

Thank you for these suggestions. The Results and Discussion have been substantially rewritten to create more logical flow, sharpen arguments, relocate some material, and thus be more distinct in their contents.

6. *The discussion section still has more areas of the findings yet to be discussed. The findings from heuristic framework and equity framework can be discussed to conclude on the varying degree of public health impacts of those components in five exemplar countries. Similarly, it can be linked with significance of these findings at the end.*

The Discussion has now been completely rewritten to focus on the three knowledge gaps identified in the Introduction and the significance of addressing them through the findings.

Reviewer 3

1. *Authors' efforts in bringing together this important piece of evidence are commendable. The manuscript presents an important review and documentation on the use of quarantine as an international travel measure during the COVID-19 pandemic, with a comparative analysis of its implementation and equity impacts across five “exemplar” countries. The review is relevant.*

We thank the reviewer for recognizing the paper’s contribution to lesson learning for this specific type of travel measure through comparative analysis. We are not aware of another study of this kind. We are especially concerned at the lack of attention since the pandemic to ITMs which has become, as one WHO director put it, a “sleeper issue” until the next pandemic.

2. *However, the manuscript is too lengthy and would benefit from concise revisions to improve overall readability and clarity. Consider making it more concise to enhance readability and alignment with journal guidelines.*

We acknowledge that the paper is long and have made efforts to reduce its length through further editing for improved conciseness and readability. For example, we have eliminated Table 1, suggest moving all tables to annexes, and cut text throughout. Almost 1000 words have been cut.

3. *Several tables and supplementary information can be moved to an appendix or online supplementary materials.*

We are happy for most, if not all, tables to be moved to annexes as on-line supplementary materials. Indeed, we originally envisioned that this would be the case. We have indicated the small number of tables and boxes that we think should remain as part of the text.

4. *Abstract: The abstract lacks clarity, particularly in describing the methodology and key findings. The type of study, Data sources, Key results, and Main conclusions should be clearly stated.*

We have rewritten the Abstract to reflect the contents of the substantially revised paper. The Abstract is not a structured abstract (e.g., Rationale, Methods, Findings, Conclusions) but we have sought to provide this information more clearly as suggested.

5. *Introduction and Background: The distinction between the Introduction and Background is unclear. Please refer to the journal’s author guidelines: if a separate background section is required, ensure it has a distinct focus; otherwise, consider merging it with the introduction. The “Background” section title may be changed to something more specific or merged as needed.*

Based on this comment, and Reviewer #2 comments #1 and #2, we have rewritten the Introduction accordingly. This includes incorporating most of the Background into the Introduction and reducing duplicate text.

6. *Methods: The methods section lacks detail. What specific methodology was used? While secondary data use is mentioned, the process of data collection, analysis framework, and ethical considerations needs to be stated clearly. Clarify the study design. What type of review is this? Terms such as “exemplar countries” and “eight variables” should be briefly clarified. What criteria defined “exemplar” status?*

As described in our response to Review 2, comment #3, we have rewritten the Approach and Methods section to flow more logically (data collection, case study selection, framework for comparative analysis, approach to assessing equity, and research ethics approval) and provide more detail throughout as requested. The criteria defining exemplar status was previously defined as “the effective use of quarantine measures to reduce international travel-related SARS-CoV-2 introductions and onward transmission” as identified in different sources identified. The reorganization of this section should hopefully make this more clear.

7. *Results: The presentation of results is too descriptive in places. Data should be referenced and dated precisely (e.g., periods of quarantine, policies used). The condition of quarantine must be compared to standard guidance or relevant country-specific policies, including references.*

We have now rewritten the results to be less descriptive and more analytical. We have sought to reference policies that are used as examples. However, one of the major challenges of this subject is that policies changed frequently and were not well documented publicly. None of the countries had official chronologies to draw upon. Dates when quarantine policies were announced, enacted, and lifted were often missing or even contradictory. The compiling of the data in the now Annexes was thus a significant task. Referencing data and dates precisely remains a major challenge. Nonetheless, we believe that these tables are the most comprehensive datasets currently available on quarantine use in the five countries.

8. *Page 47: The photo included needs a source and context. Where was this taken? Is permission required?*

This photograph has now been removed. The source was a contact of one of the authors but upon reflection it is not necessary to include in this paper.

9. *Tables 3–9 are too long and hinder the manuscript’s flow. Consider moving some to supplementary files.*

The information in these tables took a substantial effort to compile and thus unique in their composition. As such, we believe that they offer a unique dataset for future research. Nevertheless, we agree that these tables can be better located as on-line supplementary annexes for reference purposes only.

10. *Tables 4, 5, 6, 7, 8, and 9 should be made more concise and reader-friendly, or can be moved to supplementary files.*

See above comment.

11. *Discussion and Conclusion: Ensure the discussion reflects how the findings contribute to the current knowledge. Link findings more directly to the literature and to policy/practice implications. Keep the conclusion concise, and ensure it clearly summarises the key takeaways.*

The Discussion has now been completely rewritten to focus on the three knowledge gaps identified in the Introduction and the significance of addressing them through the findings. The Conclusion has been revised too to be more concise in providing key takeaways.
